# A qualitative study of overdose responses among Chicago IDUs

**DOI:** 10.1186/1477-7517-5-2

**Published:** 2008-01-24

**Authors:** Susan G Sherman, Donald S Gann, Gregory Scott, Suzanne Carlberg, Dan Bigg, Robert Heimer

**Affiliations:** 1 Department of Epidemiology, Johns Hopkins Bloomberg School of Public Health, 615 N. Wolfe Street, E6543, Baltimore, MD 21205, USA; 2 Department of Sociology, Depaul University, 990 W. Fullerton Ave., Ste. 1100, Chicago, IL, 60614, USA; 3 Chicago Recovery Alliance, 400 E Ohio Street – Suite 3103, Chicago IL 60611, USA; 4 Department of Epidemiology and Public Health, Yale School of Medicine, PO Box 208034, 60 College Street, New Haven, CT 06520-8034, USA

## Abstract

**Background:**

Opioid overdose is a leading cause of death among injection drug users. Over half of injection drug users report at least one nonfatal overdose during their lifetime. Death from opioid overdose rarely occurs instantaneously, but rather over the course of one to three hours, allowing ample time for providing life-saving measures. In response to the prevalence of overdoses in the U.S., there are a growing number of overdose prevention and naloxone distribution programs targeting the injection drug using community.

**Methods:**

We explored injection drug users' experiences with opioid overdose response, examining differences between overdose responses in which naloxone was and was not used. The current study is based upon qualitative interviews (N = 31) with clients of the Chicago Recovery Alliance needle exchange program who had witnessed an overdose in the past six months. The interviews explored participants' drug use history, personal overdose experiences, and details concerning their last witnessed overdose. Verbatim transcripts were coded and analyzed thematically to address major study questions.

**Results:**

Participants were 81% were male, their median age was 38. They reported having injected a median of 10 years and having witnessed a median of six overdoses in their lifetime. All described overdoses were recognized and responded to quickly. None of the overdoses resulted in a fatality and naloxone was successfully administered in 58% of the last witnessed overdoses. Administering naloxone for the first time was characterized by trepidation, but this feeling dissipated as the naloxone quickly took effect. Emergency medical personnel were called in 10 of the 31 described overdoses, including four in which participants administered naloxone. The overwhelming majority of experiences with police and paramedics were positive

**Conclusion:**

Overall, our small study found that the overdose prevention efforts build on extensive knowledge possessed by IDUs. Teaching IDUs how to use naloxone is an effective risk reduction strategy.

## Background

Opiate overdose is the single greatest cause of mortality among IDUs in the U.S. [1]. Over half the deaths of heroin injectors are attributed to opiate overdoses [2], far exceeding the proportion of deaths due to AIDS or other morbidities. As a result, IDUs have an annual mortality rate of 2%, a rate six to twenty times higher than their non-drug using peers [2]. In addition to the burden of overdose mortality, IDUs suffer a high prevalence of non-fatal opiate overdose. Studies show the proportion of heroin IDUs reporting at least one non-fatal overdose in their lifetime was 48% in San Francisco [3], 42% in New York City [4], 38% in London [5], and 68% in Sydney, Australia [6].

Death from opioid overdose rarely occurs instantaneously, but rather over the course of one to three hours, allowing ample time for providing life-saving measures [7,8]. Commonly referred to by its trade name, Narcan, naloxone is an opioid antagonist routinely used by emergency medical personnel to reverse opiate overdose. Naloxone has no pharmacological effects in the absence of opioids and is a cost-effective tool for preventing death and disability from opioid overdose [9]. In the U.S. as elsewhere, naloxone dispensation is regulated as an unscheduled prescription drug, meaning that it is not a dangerous drug but a prescription is required. Training drug users in appropriate overdose prevention and response can be beneficial in preventing nonfatal and fatal overdoses. Following England and Australia, a number of overdose prevention and naloxone distribution (OPND) programs have been developed in the U.S. over the past several years. Preliminary pilot evaluations have demonstrated feasibility and acceptability among IDUs [3,10-12]. The current study explores what informed IDUs' choice in overdose response, comparing responses that did and did not include naloxone administration.

## Methods

### Training

CRA has operated a syringe exchange program and conducted HIV prevention outreach with drug users since 1991. An average of 340 drug users are reached weekly through 16 mobile van and storefront sites. In 1998, a volunteer physician began prescribing and dispensing naloxone from some of CRA's busier outreach sites, the first in the U.S. In 2000, CRA expanded these overdose prevention efforts to all sites. All CRA staff were trained by a physician in an extensive curriculum that included: basic opioid neurophysiology; opioid and opioid antagonists pharmacodynamics; overdose risk factors, prevention, and symptoms; aspiration prevention; rescue breathing; naloxone administration; and dosing guidelines.

Trained CRA staff implemented the 30-minute OPND intervention focused on overdose prevention techniques, overdose recognition, and response options. Trainings occurred in small groups or one-on-one. All CRA participants were offered the training, regardless of injection drug use status. After participants received the training, staff collected a brief medical history and administered a brief post-training checklist that tested participants' retention of the training. Participants then received a 10 ml multi-dose vial of naloxone, three sterile intramuscular syringes, a pocket-sized overdose response and recognition card, and a naloxone prescription.

### Study population and data collection

Purposive sampling was used to recruit IDUs who exchanged syringes at three of CRA's sites. Eligible participants were 18 years or older and had witnessed an overdose within the past six months. A qualitative researcher conducted interviews twice weekly over the course of three months in 2004. During these times, CRA staff referred individuals to the interviewer if they had reportedly witnessed an overdose within the previous six months. This was ascertained in one of several ways: they requested a naloxone refill; they asked to receive OPND training; or through casual conversation with staff. The researcher further verified both study criteria.

If clients seemed relatively articulate for the purposes of a qualitative interview provided informed consent, the researcher conducted individual semi-structured qualitative interviews in her car so that confidentiality could be maintained. The qualitative interview guide explored: illicit drug use history; overdose experiences; lessons learned from the OPND training (if appropriate); and details concerning their last witnessed overdose. Interviews ranged in length from 30 to 45 minutes and were tape-recorded and transcribed. The study was found exempt from review by the Johns Hopkins Bloomberg School of Public Health Committee on Human Research and the Yale School of Medicine Human Investigation Committee. Respondents received $20 compensation for their participation.

### Data analysis

Two qualitative researchers analyzed the data thematically in a multi-step process using the constant comparative method that is central to grounded theory [13]. After reading several interviews for comprehension of interview content, five interviews were chosen for open coding. Open coding is a process of reading small segments of text at a time and making notations in the margins regarding content or analytic thought, without being constrained by existing theoretical explanations [13]. The labels applied in the open coding process were then synthesized into a code list to remove redundancy and similar labels were grouped together. The initial theme list closely followed the in-depth interview instrument as it was quite detailed in terms of lines of questioning and related probes. The code list was then used to code all of the interviews. Data were entered into Atlas-ti version 4.2, a qualitative data management program, in order to organize all project coding and memos.

## Results

Participants (N = 31) were 81% were male and the median age was 38, ranging from 21 to 55 yeas old. Participants reported having injected a median of 10 years and 81% injected daily. Participants reported having witnessed a median of six overdoses in their lifetime and 32% had witnessed at least one fatal overdose in their lifetime. None of the overdose events described in this analysis were fatal. Twenty-two participants had received OPND training, but only 18 administered naloxone in the described events.

### Overdose response

Participants considered a number of factors in deciding how to respond. Fear of legal consequences weighed heavily, but was counterbalanced by a desire to safe a life.

### Combination of responses

A range of additional revival methods were reported: 10 participants called 911, including four instances when naloxone was administered; six poured cold water or ice on the person, including two instance when naloxone was administered; six hit the person, including three instances when naloxone was administered; three rubbed the person's sternum, including two instances when naloxone was administered; and four administered mouth-to-mouth resuscitation. In five cases, the overdose survivor was taken to the hospital, including twice when naloxone was administered.

### Use of naloxone

All of the 18 participants who had administered naloxone had been through the CRA training. In all these cases, naloxone effectively revived the victim. Participants reported that they had been present at a median of two overdose events in which naloxone had been used. In describing the first time that they administered naloxone, key themes were that of trust in oneself and naloxone.

*I did think of the Narcan but I didn't want to use it because I wasn't sure if I administered it but I didn't do it correctly, or if I'd have killed him ... And then I thought, well, maybe if he dies and I didn't do anything... So, I injected him once with it..." *(40 year old woman)

After using naloxone the first time and seeing rapid and positive results, participants gained a sense of comfort.

*When I know what's happening I feel pretty comfortable now. As long as there's Narcan somewhere in the house, I feel like I can deal with it. I get really scared, of course, and start panicking but I don't panic in a way that' s making me not think straight*. (44 year old man)

Advance planning is an important step in having naloxone accessible and available when needed, as emphasized in the CRA training. A young man who lives with several IDUs described the accessibility of naloxone in their house.

*We keep it in needles in the house. We keep it all ready... They give us special needles, larger ones for muscles. We keep one of those in every room of people who use and in the bathroom*. (27 year old man)

Participants were overwhelmingly pleased with their ability to administer naloxone during an overdose. As one 55 year old man said, "You can save someone and you don't have to worry about people dying on you anymore. I've had lots of friends die. Now you know that you can save 'em if you have narcan."

### Revivals without naloxone

Thirteen participants did not use naloxone in response to the overdose. In absence of the drug, numerous responses were employed successively until revival. A 26 year old man's description is typifies this scenario.

*I knew something's wrong so I start slappin' him and tryin' to wake him up and he wasn't waking up, so...then I drove to the Hospital and I pulled into the parking lot. I didn't want to do it because, for one, I was high as hell myself and I didn't want to have to bring him in there with a car full of heroin... For 10 minutes he was startin' to get very cold and his face was turnin' blue and I took water and I threw it in his face and he kind of moved around a little bit so I slapped him in his face and started shakin' him and he finally came out of it*.

A 36 year old woman tried CPR before calling 911 when her friend overdosed in her bathroom. She took the CRA training a month after this occurred.

*A girlfriend of mine went into the bathroom and she come out and like five minutes later she fell out on the floor and turned blue. CPR wasn't working so we had to call 911. They came and gave her the injection of narcan and it brought her out of it*.

One participant who had naloxone on hand decided to revive his friend through mouth-to-mouth resuscitation because she was afraid of using the naloxone. She was unsure if it would work.

*Well first I went to him and I started mouth to mouth like I always do because I see the pale face and that scared me. I was just focusing on him breathin' and he started breathing again, and every time I gave him a breath of air, he started breathing again*. (44 year old man)

### Factors influencing contacting emergency personnel

Participants described a range of thoughts and emotions that influenced their decision to call 911. When asked about this decision process, participants unanimously began their response with expressing their fear of police accompaniment and the possibility of arrest. Past negative experiences with medical personnel, albeit less frequently reported than fear of police, was also mentioned as a barrier. The following excerpts describe participants' struggle between calling 911, their desire to help their friends, and other issues of issues.

*I'm thinkin', 'Oh, my god, I'm going to jail. Oh, my god, my friend's gonna die.' I'm worried about him, I don't want him to die, but yet I'm looking at the legal aspect. I got, like, 20 bags of dope in the car. I don't want to go to jail*. (39 year old man)

*What am I gonna tell the cops? What am I gonna tell my mom? ...All those questions that are runnin' through your head. What's gonna happen? Are we all gonna get arrested because of this? Is he gonna die? What am I gonna tell his parents? All sorts of crazy shit, because I've known him for a long time and thought, if this happens with me and then his family's gonna blame me. I had all these things going through my head but my main concern was tryin' to get him to wake back up*. (42 year old man)

One man who did not have naloxone available felt he had no choice but to call 911 because that was the best way to help his friend.

*I'd rather have the person live. I really couldn't live with myself if I didn't call and the guy died. I had the chance to call them and he would live. I wouldn't be able to survive, you know, that would really bug me*. (44 year old man)

### Paramedic's reactions to recent overdose

Half of those who called 911 reported positive interactions with emergency responders. A participant who both administered naloxone and called 911 received positive feedback.

*Well, when the ambulance got there I said, 'You better give him a little more [naloxone], man.' They asked me about how I got naloxone and I told them about the training...they said I did a good job*. (38 year old man)

But a few did have negative experiences with paramedics either in the way in which they were treated or expressed doubt at participants' ability to administer naloxone.

*I told them what I did and they were kind of nasty to me. They said that I shouldn't have administered something, that I'm not trained in it, that I'm not a paramedic. I said, 'Well, what should I do, let him die?' That's when he told me I wasn't – he kinda blew on me about that*. (55 year old man)

Police accompanied paramedics in four instances, resulting in no arrests and no negative interactions. As reported by a 44 year old man, "The police were pretty cool. I told him what I did and he said, 'You probably saved his life. He was cool about it."'

## Discussion

The current study is the first to qualitatively explore how IDUs' responded to an overdose and their feelings about their choices in the context of an overdose prevention and naloxone distribution program. Regardless of the responses employed, results demonstrate individuals' desire to and ability to help their peers. Naloxone was well received and utilized by the majority of those trained. Any fear of initial use dissipated quickly as naloxone took effect, although fear did prevent one participant from using the naloxone. Fear of administering naloxone for the first time should be addressed in OPND trainings. OPND trainings build upon participants' extensive overdose experience. Ultimately, participants were proud to be able to save someone's life.

Although rarely reported, several participants reported having performed incorrect responses to overdose, such as pouring ice or hitting on the victim. Participants' reliance on such methods will hopefully diminish over time and as they have seen naloxone in use. When participants return for their naloxone refills, it is important for staff to ask questions about overdose response and address these types of myths and any other issues that arise.

Administering naloxone is a component of the caretaking role that many IDUs have within their injection networks [14]. Naloxone distribution programs build on IDUs' natural helping roles and extend the benefits of such risk reduction programs beyond their hours of operation and their geographic location. In the context of general resistance to interface with both hospital and legal/law enforcement institutions, such trainings provide a positive alternative. Of course the inherent downside to this self care is the potential for increasing IDUs' distance from "legitimate" health care services. But OPND trainings are an opportunity to address the importance of both self care and promoting the use of and referrals to important health and social services.

OPND trainings can help people respond appropriately to overdose events when they are wary of calling 911. The extent of this fear and its effects on overdose response has been documented in several studies [6-15]. We found that 35% of participants did call 911 although extensive fear of police accompaniment was expressed. Several positive and no negative police interactions were reported and most interactions with paramedics were positive. Educating first responders, such as emergency medical personnel and police, about overdose prevention programs is an important component of OPND programs and could reduce negative experiences between overdose bystanders and first responders.

The current study is subject to several limitations. The analysis is not generalizable beyond this small sample of CRA participants. As characteristic of many qualitative studies, the sample was not randomly; rather, individuals were selected based not only on inclusion criteria but also on the basis of judgements about individuals' ability to articulate their experiences. Lastly, all data collection was completed prior to analysis, which limited the ability to explore emergent themes as they arose during "real time [16]."

The study points to needed future research. As OPND programs continue to be established and existing programs grow in the U.S., long-term and more rigorous research is needed. Although existing research is favourable, larger studies with longer-term outcomes are needed to truly establish effectiveness in the far-reaching goal of reducing fatal opiate overdose rates. At this time, only one such longitudinal study of has been conducted in Australia and results indicated OPND training was associated with overdose reductions [17].

The current study is the first qualitative study among a small body of research that evaluates OPND programs. The study indicates the potential benefits of these programs and underscores the need for more rigorously designed evaluations. In this small sample, we found that participating in the OPND training provided IDUs with a sense of dignity – both through being educated about such a salient issue in their lives and having the opportunity to intervene safely and effectively to save someone's life. Overdose prevention education and naloxone distribution provide IDUs with the tools to be effective first responders. OPND trainings not only provide the necessary tools for effective overdose response to reduce fatal overdoses but also provide essential prevention information that could help to reduce the prevalence of all overdoses.
